# Sex-Specific Associations of Diabetes With Brain Structure and Function in a Geriatric Population

**DOI:** 10.3389/fnagi.2022.885787

**Published:** 2022-06-28

**Authors:** Elias G. Thomas, Hanneke Rhodius-Meester, Lieza Exalto, Sanne A. E. Peters, Liselotte van Bloemendaal, Rudolf Ponds, Majon Muller

**Affiliations:** ^1^Department of Internal Medicine, Geriatrics Section, Amsterdam Cardiovascular Science, Amsterdam University Medical Centre, Amsterdam UMC, Amsterdam, Netherlands; ^2^Department of Internal Medicine, Amsterdam Public Health Institute, Amsterdam UMC, Amsterdam, Netherlands; ^3^Department of Neurology, Alzheimer Center Amsterdam, Amsterdam Neuroscience, VU University Amsterdam, Amsterdam UMC, Amsterdam, Netherlands; ^4^Department of Neurology, UMC Utrecht Brain Center, University Medical Center Utrecht, Utrecht, Netherlands; ^5^Julius Center for Health Sciences and Primary Care, University Medical Center Utrecht, Utrecht University, Utrecht, Netherlands; ^6^The George Institute for Global Health, Imperial College London, London, United Kingdom; ^7^The George Institute for Global Health, University of New South Wales, Sydney, NSW, Australia; ^8^Department of Medical Psychology, Amsterdam University Medical Centers, Amsterdam, Netherlands

**Keywords:** sex-specific analysis, brain structure, diabetes, cognitive function, vascular aging, vascular cognitive impairment and dementia

## Abstract

**Introduction:**

Globally, women with dementia have a higher disease burden than men with dementia. In addition, women with diabetes especially are at higher risk for cognitive impairment and dementia compared to men with diabetes. Differences in the influence of diabetes on the cerebral vasculature and brain structure may contribute to these sex-specific differences. We examined sex-specific patterns in the relationship between diabetes and brain structure, as well as diabetes and cognitive function.

**Methods:**

In total, 893 patients [age 79 ± 6.6 years, 446 (50%) women] from the Amsterdam Ageing Cohort with available data on brain structures (assessed by an MRI or CT scan) and cognitive function were included. All patients underwent a thorough standardized clinical and neuropsychological assessment (including tests on memory, executive functioning, processing speed, language). Brain structure abnormalities were quantified using visual scales.

**Results:**

Cross-sectional multivariable regression analyses showed that diabetes was associated with increased incidence of cerebral lacunes and brain atrophy in women (OR 2.18 (1.00–4.72) but not in men. Furthermore, diabetes was associated with decreased executive function, processing speed and language in women [B −0.07 (0.00–0.13), −0.06 (0.02–0.10) and −0.07 (0.01–0.12) resp.] but not in men.

**Conclusions:**

Diabetes is related to increased risk of having lacunes, brain atrophy and impaired cognitive function in women but not in men. Further research is required to understand the time trajectory leading up to these changes and to understand the mechanisms behind them in order to improve preventive health care for both sexes.

## Introduction

The prevalence of diabetes is increasing worldwide, with an expected rise from 537 million adults in 2021 to 783 million in 2045 (Sun et al., 2021). This not only leads to high mortality – more than 6.7 million deaths in 2021 alone – but also to high morbidity, including an increased risk of cognitive impairment and dementia (Arvanitakis et al., 2004; Yaffe et al., 2004; Liu, J. et al., 2018). However, not all individuals are similarly affected by the complications of diabetes. As early as 1979, and as confirmed more recently by cohort studies, it was shown that type 2 diabetes is a stronger risk factor for ischemic heart disease and stroke in women than in men (Kannel and McGee, 1979; Peters et al., 2014, 2020, 2021). Women with type 2 diabetes also have a higher excess risk of cognitive decline and vascular dementia, than their male counterparts, although the extent of these differences are dependent of study populations and their characteristics (Verhagen et al., 2022).

To date, it is unclear why men and women with diabetes are dissimilarly impacted by dementia. Since sex-related differences in the incidence of dementia are only present in the group with vascular dementia – not in Alzheimer's dementia – sex-specific patterns of cerebral vascular pathology may play a role in mediating these differences (Hayden et al., 2006; Chatterjee et al., 2016; Liu et al., 2018). Cerebral small vessel disease (cSVD) – including the presence of microbleeds, white matter hyperintensities and lacunes – is more prevalent in individuals with type 2 diabetes than those without (Troncoso et al., 2008; Moran et al., 2013; Geijselaers et al., 2015; Ter Telgte et al., 2018; Wardlaw et al., 2019). Although little is known about sex-specific susceptibility for cSVD, it seems plausible that the increased susceptibility of women to cerebrovascular complications of diabetes is manifested as an increased susceptibility to disease of the smaller cerebral vessels (Jiménez-Sánchez et al., 2021). In addition, diabetes is associated with increased rates of atrophy (Moran et al., 2013). Again, it is not known whether there are sex-related differences in this association, but it is known that atrophy and cSVD are closely related, and they are sometimes even collectively referred to as “brain structure” or “markers for brain health” (Mahammedi et al., 2021). Our primary goal in the present analysis is to assess if there are sex-specific pattern in the relationship between diabetes and brain structure, including cSVD and atrophy, and cognitive function as their clinical correlates.

## Methods

### Study Population

The Amsterdam Aging Cohort is an ongoing longitudinal cohort study which includes patients from the outpatient geriatric clinic at the Amsterdam University Medical Center, location VUmc (Rhodius-Meester et al., 2021). We included 893 patients with brain imaging who attended the memory clinic seeking medical care between February 2016 and June 2021. During this period, almost a patients visiting the memory clinic (89%) were willing to participate in the study. All patients were given a complete standardized comprehensive geriatric assessment (CGA) by trained nurses and doctors. This included an assessment of multiple geriatric domains, including cognition, physical function, nutrition, revision of medication in use and detailed medical history. Cognitive diagnosis – such as dementia (McKhann et al., 1984; Román et al., 1993; Neary et al., 1998; McKeith et al., 2005; Dubois et al., 2007; Rascovsky et al., 2011), mild cognitive impairment (MCI) (Albert et al., 2013), or subjective cognitive decline (SCD) (Studart and Nitrini, 2016) – were evaluated in a multidisciplinary consensus meeting. Our analysis included only patients who underwent brain Magnetic Resonance Imaging (MRI) or Computed Tomography (CT) as part of the diagnostic work-up. All patients gave written informed consent for their data to be used and the study was approved by the local Medical Ethics Committee.

### Cardiovascular Risk and Disease

Diabetes mellitus (DM) was defined either as having a history of diabetes or using antidiabetic medication. Other cardiovascular diseases – including coronary disease, heart failure, atrial fibrillation, and peripheral artery disease – were assessed on the basis of medical history and double checked with the patient and their family or carer. We dichotomized smoking status (never smoked v. ever smoked). Blood pressure and gait speed, as well as patient height and weight, were measured during the visit (Odden et al., 2012). Venous blood was drawn from all patients to measure cholesterol levels and non-fasting glucose. Medication as provided by the patient's pharmacy was reviewed with the patient, and with a partner, family member or carer if necessary.

### Cerebral Small Vessel Disease

Brain imaging was performed during a patient's first visit using CT (*n* = 238), 1.5T MRI (*n* = 162) and 3T MRI (*n* = 478) devices. The scans were reviewed and scored visually by two trained experts supervised by a clinical radiologist. Atrophy was scored on T1 sequence using visual rating scales ranging from 0 to 4 for medial temporal lobe atrophy (MTA), and from 0 to 3 for global cortical atrophy (GCA) (Harper et al., 2015). The average of the left and right side was used for MTA. White matter hyperintensities were scored on FLAIR/T2 sequence using the Fazekas scale (0–3) (Fazekas et al., 1987), and the number of microbleeds (on susceptibility-weighted imaging) and lacunes were counted. In this manuscript, we refer to either brain atrophy, white matter hyperintensities, lacunes or microbleeds collectively as “brain structure abnormalities.”

### Cognitive Function

To assess whether the observed differences in brain structure also had a functional impact, cognition was included in the analysis. Cognitive performance was assessed in a standardized manner by trained neuropsychologists and divided into four domains: memory, language, executive function, and processing speed. All patients were assessed using the Mini Mental State Examination (MMSE) and the Geriatric Depression Scale (GDS). Memory was tested with the auditory verbal learning test (Van Der Elst et al., 2005) and Visual Association Test (VAT) (Lindeboom et al., 2002). Language was tested using the Category Fluency Animals Test (Van Der Elst et al., 2006) and the VAT naming test, a component of the VAT. Processing speed was examined with the Stroop Color-Word test (SCWT) (Van der Elst et al., 2006) and the Trail Making Test-A (TMT-A) (Reitan, 1955). Finally, executive function was assessed with the Behavioral Assessment of the Dysexecutive Rule-changing test (BADS) (Burrell and Piguet, 2015) while correcting for speed using the SCWT and the TMT. For the purposes of the analysis, all test results were converted to Z-scores or inverse Z-scores. A higher Z-score indicates poorer performance.

### Statistical Analysis

Baseline characteristics for men, women, and the total population are reported as mean (SD), or median (interquartile range) for categorical variables. Differences between groups were analyzed using Student's *T*-test, the Mann-Whitney *U*-test, the Kruskal Wallis test, ANOVA and chi-square testing where appropriate. First, logistic regression analyses were performed to assess the association of diabetes with brain structures separately for men and women. For the logistic regression analysis, we dichotomized the scores of the visual rating scale and the values for microbleeds and lacunes. A cut-off value of two or more was used for the imaging scores of atrophy (MTA and/or CGA) and WMH (Rhodius-Meester et al., 2017). Microbleeds were dichotomized as present or not present, and a value of one or more was adopted a cut-off for lacunes (Henneman et al., 2009; Jokinen et al., 2011). Second, linear regression analyses were performed to assess the association of diabetes with cognitive functioning separately for men and women. All analyses were adjusted for age (model 1), and additionally for smoking and alcohol consumption (model 2), and hypertension and cardiovascular disease (coronary disease, heart failure, atrial fibrillation, stroke or TIA and peripheral arterial disease) (model 3). In addition, we adjusted for presence of subjective complaints, mild cognitive impairment, or dementia (data not shown). For the analyses of functional cognitive measures, furthermore, we corrected all models for level of education. A *p*-value < 0.05 was considered statistically significant. Data were analyzed with SPSS software, version 26 (IBM Corp, Armonk, NY, USA).

To determine whether male or female sex and CVD was associated with a higher risk of cSVD to a greater degree than these factors individually, we added an interaction term to the regression analysis, testing multiplicative interaction. To assess additive interaction, we calculated RERI (Relative Risk due to Interaction) (Knol et al., 2007; Knol and VanderWeele, 2012). For this analysis, when the combined risk of sex and CVD was higher than the sum of the risks associated with the individual factors, the interaction between sex and CVD was considered to constitute an additional risk factor. A RERI above zero indicated that interaction between female sex and cardiac disease had an additional effect on the outcome; a RERI below zero indicated that this was the case for the interaction between male sex and cardiac disease. In the RERI analysis, we also corrected for age, smoking, and alcohol consumption, analogous to the logistic regression analyses. These analyses were performed in R (R Core Team, 2020).

## Results

A total of 893 patients were included in the analysis (Table 1). The mean (SD) age was 79.6 years (73–86.2) and 50% were women. The prevalence of diabetes was 23.3% in men and 15.7% in women (*p* = 0.004), and men with diabetes were more often insulin-dependent compared to women (6.3% for men, 3.1% for women, *p* = 0.03). Women lived alone more often than men and their level of education was lower. Further, women had a lower prevalence of cardiovascular disease, consumed less alcohol, smoked less, had a lower body mass index (BMI) and a slightly higher diastolic BP, and they used less statins and anticoagulation drugs (Table 1). Women had slightly lower Mini-Mental State Examination scores, GDS, and men had higher brain atrophy scores. No differences in cognitive diagnosis were observed between men and women. Stratified analyses for sex and diabetes showed that differences in cardiovascular risk between men and women were more pronounced in those with diabetes compared to the total population (Supplementary Table 1).

**Table 1:** Patient characteristics stratified for sex.

	**Total**	**Men**	**Women**	***P*-value**
	***n* = 893**	***n* = 447**	***n* = 446**	
Age in years	79.6 ± 6.6	79.1 ± 6.4	79.9 ± 6.7	0.096
Living situation	487 (54.5%)	330 (74.0%)	157 (35.1%)	**<0.001**
Independent, with partner	329 (36.8%)	89 (20.0%)	240 (53.7%)	
Independent, alone	32 (3.6%)	10 (2.2%)	22 (4.9%)	
Institutionalized	45 (5.0%)	17 (3.8%)	28 (6.3%)	
Other				
Level of education				**<0.001**
Low education	180 (20.1%)	81 (18.1%)	99 (22.2%)	
Medium level education	317 (35.1%)	137 (30.7%)	176 (39.4%)	
Higher education or university	396 (44.3%	225 (50.4%)	171 (38.3%)	
Diabetes^a^	176 (19.7%)	105 (23.4%)	71 (15.9%)	0.004
Antidiabetic medication				
Oral	112 (12.5%)	69 (15.4%)	43 (9.6%)	**0.009**
Insulin	42 (4.7%)	28 (6.3%)	14 (3.1%)	**0.028**
Cardiovascular diseases				
Coronary disease	225 (25.2%)	155 (34.8%)	70 (15.7%)	**<0.001**
Heart failure	90 (10.1%)	56 (12.6%)	34 (7.6%)	0.014
Atrial fibrillation	153 (17.1%)	87 (19.5%)	66 (14.8%)	0.060
CVA/TIA	186 (20.8%)	111 (24.9%)	75 (16.8%)	**0.003**
Peripheral artery disease	33 (3.6%)	16 (3.6%)	17 (3.8%)	0.861
Cardiovascular risk factors				
Alcohol consumption in units/week	1 (0–5)	2 (0–7)	1 (0–5)	**0.003**
Smokers or ex-smokers	505 (56.6%)	289 (64.8%)	216 (48.3%)	**<0.001**
Hypertension	466 (52.2%)	228 (51.1%)	238 (53.2%)	0.525
Hypercholesterolemia	221 (24.7%)	115 (25.8%)	106 (23.7%)	0.473
Glucose in mmol/L	6.9 ± 2.6	6.9 ± 2.9	6.4 ± 2.5	0.302
BMI in kg/m^2^	25.7 ± 4.6	26.1 ± 4.2	25.3 ± 4.9	**0.012**
Systolic BP in mmHg	145.8 ± 21.8	144.5 ± 21.3	147.0 ± 22.3	0.100
Diastolic BP in mmHg	80.6 ± 10.4	79.7 ± 10.5	81.4 ± 10.3	**0.014**
LDL in mmol/L	2.56 ± 0.98	2.62 ± 0.98	2.47 ± 0.97	0.959
HDL in mmol/L	1.58 ± 0.47	1.58 ± 0.47	1.58 ± 0.47	0.101
eGFR CKD-EPI in ml/min/1.73 m^2^	67.2 ± 16.2	67.0 ± 16.4	67.4 ± 16.0	0.755
Statin use	525 (58.8%)	208 (46.6%)	160 (35.8%)	**0.001**
Blood pressure lowering agents				
Diuretics	147 (16.4%)	71 (15.9%)	76 (17.0%)	0.663
RAAS-inhibition	171 (19.1%)	90 (20.2%)	81 (18.1%)	0.434
Calcium-antagonists	63 (7.0%)	37 (8.3%)	26 (5.8%)	0.148
Beta-blockers	105 (11.7%)	61 (13.7%)	44 (9.8%)	0.075
Anticoagulation				
DOAC/VKA	168 (18.8%)	96 (21.5%)	72 (16.1%)	**0.038**
Platelet inhibition	288 (32.2%)	169 (37.9%)	119 (26.6%)	**<0.001**
MMSE	24 (21–26)	25 (22–28)	24 (21–28)	**0.008**
GDS	3 (1–5)	3 (1–5)	3 (1–5)	0.093
Brain imaging				
MTA or GCA > 2	493 (55.2%)	282 (63.2%)	211 (47.2%)	**0.007**
WMH >2	227 (25.4%)	108 (24.2%)	119 (26.6%)	0.398
Lacunes ≥ 1	204 (22.8%)	112 (25.1%)	92 (20.6%)	0.098
Microbleeds ≥ 3	163 (18.3%)	90 (20.2%)	73 (16.3%)	0.213
Cognitive diagnosis				0.085
SCD	124 (13.8%)	62 (13.9%)	62 (13.9%)	
MCI	267 (29.8%)	148 (33.2%)	119 (26.6%)	
Dementia	502 (56.2%)	236 (52.9%)	266 (59.5%)	

*Data are presented as mean ± SD, n (%) or median [interquartile range]. Differences were tested with independent t-test for continuous variables and chi-square tests for categorical and for not normally distributed continuous variables*.

*BMI, body mass index; BP, blood pressure; CVA, cerebrovascular accident; DOAC, Direct Oral Anti-Coagulant; GCA, Global Cortical Atrophy; MCI, Mild Cognitive Impairment; MTA, Medial Temporal lobe Atrophy; TIA, Transient Ischemic Attack; SCD, Subjective Cognitive Decline; VKA, Vitamin K Antagonist; WMH, White Matter Hyperintensities*.

^a^*type I or II diabetes, not specified in our data collection*.

*The bold values indicate the p values which are statistically significant*.

### Sex Differences in the Relationship Between Diabetes and Brain Structure

The sex-specific logistic regression analyses of the relation between diabetes and brain structures showed that in women, the presence of diabetes was significantly associated with an increased risk of having brain atrophy and lacunes (Table 2). Age-adjusted odds ratios were 2.16 (95% CI 1.00–4.67) and 2.60 (95% CI 1.24–2.46). However, in men, diabetes was not associated with an increased risk of having brain structure abnormalities. Additional adjustments for cardiovascular risk factors and disease (model 2 and 3) did not change these effect estimates (Table 2). Adjusting for cognitive diagnosis (subjective complaints, mild cognitive impairment, or dementia) did not change the effect estimates (data not shown). Diabetes was not associated with an increased risk of WMH and microbleeds. When adding an interaction term to the regression, we did not find a significant interaction of sex with diabetes. When assessing additive interaction using RERI analysis, a trend was seen toward an increased risk for women with diabetes of atrophy and lacunes (RERI 0.45 for the presence of atrophy and female sex, and 0.48 for the presence of lacunes and female sex) (Supplementary Table 2).

**Table 2:** The sex-specific relation of diabetes with changes in brain structure in older men and women (*N* = 893).

	**Men, *n* = 447**	**Women, *n* = 446**	**Interaction^**a**^**	***p*-value^**b**^**
	**OR (95% CI)**	**OR (95% CI)**	**OR (95% CI)**	
**Atrophy**				
Non-diabetic	Ref	Ref	Ref	
Diabetic (model 1)	1.14 (0.60–2.18)	2.16 (1.00–4.67)*	1.22 (0.50–2.94)	*p* = 0.66
Diabetic (model 2)	1.17 (0.60–2.29)	2.46 (1.11–5.42)*	1.32 (0.46–2.79)	*p* = 0.77
Diabetic (model 3)	1.00 (0.51–1.96)	2.18 (1.00–4.72)*	1.17 (0.48–2.87)	*p* = 0.71
**WMH**				
Non-diabetic	Ref	Ref		
Diabetic (model 1)	1.20 (0.76–1.89)	1.11 (0.65–1.89)	0.88 (0.47–4.00)	*p* = 0.70
Diabetic (model 2)	1.15 (0.72–1.84)	1.09 (0.63–1.88)	0.85 (0.45–1.60)	*p* = 0.61
Diabetic (model 3)	1.01 (0.63–1.62)	1.04 (0.61–1.79)	0.78 (0.41–1.49)	*p* = 0.45
**Microbleeds**				
Non-diabetic	Ref	Ref		
Diabetic (model 1)	0.59 (0.26–1.32)	0.82 (0.31–2.20)	1.04 (0.32–3.34)	*p* = 0.67
Diabetic (model 2)	0.65 (0.28–1.49)	0.86 (0.31–2.32)	0.97 (0.30–3.13)	*p* = 0.95
Diabetic (model 3)	0.46 (0.20–1.00	0.75 (0.28–2.03)	0.93 (0.29–3.01)	*p* = 0.90
**Lacunes**				
Non-diabetic	Ref	Ref		
Diabetic (model 1)	1.68 (0.92–3.04)	2.60 (1.24–2.46)*	1.10 (0.49–6.91)	*p* = 0.82
Diabetic (model 2)	1.64 (0.87–3.07)	2.72 (1.24–5.93)*	1.00 (0.41–2.10)	*p* = 0.84
Diabetic (model 3)	1.38 (0.74–2.56)	2.40 (1.13–5.07)*	0.95 (0.37–1.91)	*p* = 0.63

*WMH, White Matter Hyperintensities*.

*Data are presented as OR with (95% CI)*.

*Model 1: adjusted for age*.

*Model 2: adjusted for age, smoking and alcohol consumption*.

*Model 3: Adjusted for age, hypertension, hypercholesterolemia, and presence of CVD (coronary disease, heart failure, atrial fibrillation, CVA or TIA, peripheral arterial disease)*.

^*^p-value < 0.05.

^a^*Interaction term (sex multiplied by diabetes) added to the logistic regression*.

^b^*Statistical significance of the interaction term in the logistic regression analysis*.

### Sex Differences in the Relationship Between Diabetes and Cognitive Performance

The sex-specific linear regression analyses of the relation between diabetes and cognitive performance showed that diabetes in women was associated with a significantly lower score of executive function (beta z-score 0.07; 95% CI 0.00–0.14), processing speed (beta 0.06; 95% CI 0.90–0.95), and language (beta 0.07; 95% CI 0.01–0.12) (Table 3). In men, diabetes was not associated with cognitive performance. Additional adjustments for cardiovascular risk and disease, and cognitive diagnosis did not change the effect estimates (data for cognitive diagnosis not shown). We observed an interaction of sex and diabetes: women with diabetes were at increased risk for impaired processing speed (B 0.17 (0.03–0.33), *p* = 0.04). We observed no interaction between diabetes and sex in the other cognitive domains.

**Table 3:** The sex specific relation of diabetes with cognitive performance in older men and women (*N* = 893).

	**Men**	**Women**	**Interaction^**a**^**	***p*-value^**b**^**
	**B (95% CI)**	**B (95% CI)**	**B (95% CI)**	
**Memory**				
Non-diabetic	Ref	Ref	Ref	
Diabetic (model 1)	0.02 (−0.09 to 0.03)	0.02 (−0.07 to 0.03)	0.09 (−0.26 to 0.46)	0.59
Diabetic (model 2)	0.03 (−0.09 to 0.02)	0.02 (−0.07 to 0.03)	0.10 (−0.26 to 0.47)	0.57
Diabetic (model 3)	0.03 (−0.08 to 0.02)	0.01 (−0.06 to 0.03)	0.10 (−0.27 to 0.47)	0.59
**Executive function**				
Non-diabetic	Ref	Ref		
Diabetic (model 1)	−0.02 (−0.09 to 0.04)	−0.07 (−0.14 to 0.00)*	−0.04 (−0.36 to 0.28)	0.81
Diabetic (model 2)	−0.02 (−0.09 to 0.04)	−0.06 (−0.13 to 0.01)	−0.03 (−0.32 to 0.32)	0.98
Diabetic (model 3)	−0.01 (−0.06 to 0.06)	−0.07 (−0.13 to 0.00)*	−0.01 (−0.33 to 0.32)	0.97
**Processing speed**				
Non-diabetic	Ref	Ref		
Diabetic (model 2)	−0.03 (−0.08 to 0.01)	−0.06 (−0.10 to 0.02)*	0.19 (0.03 to 0.34)	**0.02**
Diabetic (model 3)	−0.03 (−0.08 – 0.02)	−0.06 (−0.10 to 0.01)*	0.18 (0.02 to 0.33)	**0.03**
Diabetic (model 1)	−0.02 (−0.07 to 0.01)	−0.06 (−0.10 to 0.02)*	0.17 (0.03 to 0.33)	**0.04**
**Language**				
Non-diabetic	Ref	Ref		
Diabetic (model 1)	−0.01 (−0.06 to 0.04)	−0.07 (−0.12 to 0.01)*	−0.18 (−0.53 to 0.17)	0.31
Diabetic (model 2)	−0.01 (−0.05 to 0.07)	−0.06 (−0.12 to 0.00)	−0.12 (−0.48 to 0.22)	0.14
Diabetic (model 3)	0.00 (−0.05 to 0.04)	−0.07 (−0.012 to 0.01)*	−0.14 (−0.49 to 0.21)	0.43

*Data are presented as unstandardized B with (95% CI)*.

*A negative B signifies a correlation with worse Z-scores, e.g. with worse cognitive performance*.

*Model 1: adjusted for age and level of education; Model 2: adjusted for age, level of education, smoking and alcohol consumption; Model 3: Adjusted for age, level of education, presence of CVD (coronary disease, heart failure, atrial fibrillation, CVA or TIA, peripheral arterial disease), hypertension and hypercholesterolemia*.

^*^p-value linear regression < 0.05.

^a^*Interaction term (sex multiplied by diabetes) added to the logistic regression*.

^b^*Statistical significance of the interaction term in the logistic regression analysis*.

*The bold values indicate the p values which are statistically significant*.

## Discussion

In this study of 893 patients attending a geriatric outpatient memory clinic, we found that the presence of diabetes was associated with an increased risk of having brain structure abnormalities, specifically lacunes and atrophy, in women but not in men. We also found an additive interaction between female sex and these brain structure abnormalities, as tested by a RERI analysis. This finding complements previous studies which showed that women with diabetes may be more at risk of multiple forms of vascular pathology than men with diabetes, including coronary heart disease, stroke, and vascular dementia (Huxley et al., 2006; Peters et al., 2014; Chatterjee et al., 2016). It could be argued that other age-mediated cardiovascular risk factors such as hypertension and cardiac disease play a role in mediating the relationship between diabetes and brain structure abnormalities. However, we show that this relationship was independent of age, lifestyle, cardiovascular risk factors, and cardiac disease.

Additionally, we found that diabetes was significantly associated with worse cognitive performance in terms of executive function, processing speed and language, in the women in our population but not in the men. An interaction between sex and diabetes was also observed for processing speed, further strengthening the hypothesis of a true sex difference. These findings are in line with a recent study showing that women with diabetes have a higher risk of accelerated cognitive decline than men with diabetes (Verhagen et al., 2022). Sex-dependent physiology, as well as socio-cultural differences between men and women, may be the cause of these differences. We postulate a number of hypotheses below which may explain the association between diabetes and brain structure abnormalities as it is seen in women but not in men.

### Pathophysiological Differences

Mechanisms which may affect susceptibility to the vascular complications of diabetes include altered coagulation, oxidative stress, endothelial dysfunction and impaired vasodilation (Kautzky-Willer et al., 2016; de Ritter et al., 2020). Women with diabetes might be in a more pro-thrombotic state than men, which may lead to lacunes and atrophy, and a more general decline in brain health, even when the prevalence of diabetes is similar in both sexes (Smith et al., 2012; Neergaard-Petersen et al., 2014). They generally also have greater levels of systemic inflammation and more oxidative stress than men with diabetes, leading to impaired vascular reactivity, which is specifically associated with the occurrence of lacunes (Mrgan et al., 2018). Sex-dependent differences in vascular physiology may therefore render women more susceptible to the cerebrovascular complications of diabetes, and also lead to functional decline.

Levels of central adiposity in men and women with diabetes may also be of importance. There is evidence to suggest that women have a poorer cardiovascular risk profile than men when they are diagnosed with diabetes, especially when central adiposity is measured (Paul et al., 2012; Peters et al., 2016a). This may be the result of a longer period of development of diabetes in women: women are more insulin-sensitive in middle age, and their insulin sensitivity deteriorates more than in men before they reach the diagnosis of diabetes. A longer period of time before a formal diagnosis of diabetes can be made may also lead to an increased occurrence of other risk factors such as abdominal adiposity, and to higher levels of subclinical damage mediated by hyperglycemia (Woodward et al., 2015; Peters et al., 2016b). Abdominal adiposity is independently related to brain structure abnormalities, including silent lacunary infarcts, and therefore may mediate the increased prevalence in women with diabetes by comparison with men (Yamashiro et al., 2014).

Furthermore, there is little awareness in the field of geriatrics about the long-term cardiovascular effects of pregnancy-related complications, as well as other women-specific factors such as timing of menopause and gestational diabetes (Keskin et al., 2015; Kuh et al., 2018). These important cardiovascular parameters are therefore often not registered in medical files, as is the case in our dataset (Wilkins-Haug et al., 2015). It has long been known that menopausal status and the timing of menopause influence cardiovascular risk, abdominal obesity, occurrence of DM, and clinical course of dementia (Archer, 2009; Gong et al., 2021; Hickey and Mishra, 2021). They may therefore also have affected the clinical outcome in our study. Further studies investigating the long-term effects of DM should therefore include sex-specific cardiovascular risk factors to assess their impact on brain structure and cognitive function.

### Sex-Related Differences in Current Care

As a consequence of ongoing lower inclusion rates of women in studies investigating the long-term effects of diabetes, at least in part, it is unclear which mechanisms lead to the poorer clinical outcome of women with diabetes (Norhammar and Schenck-Gustafsson, 2013). However, there are several scientific findings which may play a role. Social gender norms have a profound influence on patients' disease perception, moment of referral, interpretation of symptoms and the likelihood of receiving guideline-recommended treatment. In the case of diabetes, evidence shows that women diagnosed with diabetes attain glycemic targets less often, and are screened less for the complications of diabetes (Choe et al., 2018). Risk factor targets for co-morbid cardiovascular disease are also less often achieved in women (Ferrara et al., 2008; Wannamethee et al., 2012; Rossi et al., 2013; de Jong et al., 2020). Since early intervention in diabetes improves long-term outcome, this may also have implications for the incidence of (cerebral) complications (Group UPDS, 1998). The same truth holds for cognitive impairment: women with dementia are generally referred later than men (Howard et al., 1998; Sourial et al., 2020). Women might therefore experience delay in receiving adequate supporting care when experiencing cognitive impairment. Hence, inequities in the recognition and treatment of (cardiovascular) risk factors for dementia, as well as the recognition and treatment of dementia itself, may play a role in the occurrence of sex-related differences in the cerebral complications of diabetes.

### Strengths and Limitations

A major strength of our study is the standardized work-up of a large group of “real-life” patients. We included a multi-domain assessment which was part of medical routine care. Because of this integration in regular care practice, routinely used measurements and tools were used, facilitating the translation of our research to clinical practice. In addition, we combined these clinically used parameters with imaging markers as well as extensive neuropsychological testing, bridging the gap between etiological research and clinical practice. Our study has also several limitations. Firstly, because of our cross-sectional design, we cannot draw conclusions about the causality of our findings. We balanced this with logistic regression models in which we corrected for confounding factors. Related to this, we have no data showing possible differences between brain structure at the time of diagnosis of diabetes, and later life. It is possible that women with diabetes already have worse brain health at this time, and that it is not a direct consequence of diabetes, but merely coexists due to other pathological processes. Furthermore, HbA1c and time since diagnosis of DM were not included in our dataset. We were therefore unable to assess the influence of glycemic regulation. We did include non-fasted glucose, which was similar for men and women, in our baseline characteristics. Also, it is remarkable that the mean age in our sample is similar for men and women. Since the life expectancy in women exceeds the life expectancy in men, we would expect a higher mean age for women. The similar age for men and women may reflect underlying gender bias in referral to our clinic. However, it may also be explained by other forms of sampling bias not related sex or gender. Furthermore, as mentioned before, women-specific pathology was hardly registered at all in our patient files. A history of gestational diabetes, polycystic ovary syndrome and premature menopause contribute to the excess risk of diabetic complications and diabetes (Soedamah-Muthu et al., 2004; Huxley et al., 2006; Peters et al., 2014). Furthermore, preeclampsia is related to structural brain damage later in life (Siepmann et al., 2017). Lastly, premature menopause is additionally associated with poorer cognitive performance and a higher risk of dementia later in life (Ryan et al., 2014). However, the precise relationship between cerebrovascular disease and pregnancy-related cardiovascular disease remains unclear, since a recent review concluded that gestational hypertension is not related to cerebral stroke (Lo et al., 2020). Finally, other factors that affect the social position of patients and therefore their quality of care – such as class, cultural background, and variables associated with poorer referral and poorer health care provision in general – were not available in our study (Vaccarino et al., 2002; LaVeist et al., 2003).

In conclusion, this sex-specific analysis of the association between diabetes and its cerebrovascular complications shows that diabetes is significantly associated with brain structure abnormalities and function in women but not in men. We can only speculate on the nature of these differences, and whether they are dependent on gender or sex. Although we did not find a statistically significant interaction between female sex and diabetes, the differences in associations for men and women are striking and underline the importance of the sex-specific analysis of clinical data. Further research should at least include data on abdominal obesity and female-specific risk factors such as pregnancy-related complications and menopause, and more studies are needed to elucidate the mechanisms which contribute to the association between diabetes, brain structure and cognition in women. Elucidating the sex-specific relationships between diabetes and cSVD may help to understand the gap in burden of dementia and help to achieve more equity in the care for this group of patients.

## Data Availability Statement

The data analyzed in this study is subject to the following licenses/restrictions: The data is not publicly available since it contains clinical privacy-sensitive patient information. Requests to access these datasets should be directed to majon.muller@amsterdamumc.nl.

## Ethics Statement

The studies involving human participants were reviewed and approved by Medical Ethical Committee of the Amsterdam UMC, location VUmc. The patients/participants provided their written informed consent to participate in this study.

## Author Contributions

Study conception and design and data collection: ET, MM, and HR-M. Analysis and interpretation of the results: ET, MM, HR-M, and SP. Draft manuscript preparation: ET, MM, HR-M, LE, SP, RP, and LB. All authors reviewed the results and approved the final version of the manuscript.

## Funding

HR-M was recipient of the Memorabel Dementia Fellowship 2021 (ZonMw project number 10510022110004). Also, we acknowledge the support from the Netherlands CardioVascular Research Initiative: the Dutch Heart Foundation (CVON 2018-28 and 2012-06 Heart Brain Connection), Dutch Federation of University Medical Centres, the Netherlands Organisation for Health Research and Development, and the Royal Netherlands Academy of Sciences.

## Conflict of Interest

HR-M performs contract research for Combinostics. All funding is paid to her institution. The remaining authors declare that the research was conducted in the absence of any commercial or financial relationships that could be construed as a potential conflict of interest.

## Publisher's Note

All claims expressed in this article are solely those of the authors and do not necessarily represent those of their affiliated organizations, or those of the publisher, the editors and the reviewers. Any product that may be evaluated in this article, or claim that may be made by its manufacturer, is not guaranteed or endorsed by the publisher.
