# Supplementary material for: Sex-Specific Associations of Diabetes With Brain Structure and Function in a Geriatric Population
Source: Front Aging Neurosci. 2022 Jun 28;14:885787. doi: 10.3389/fnagi.2022.885787 (PMC9273850; doi:10.3389/fnagi.2022.885787)
Supplement: Supplementary file 1 [file Data_Sheet_1.PDF]

Supplementary Material

Suppl Table 1 Patient characteristics stratified for sex in patients with diabetes

	**Diabetes**	
	Men (n=105)	Women (n=71)
Age in years	77.8 ± 6.4	79.2 ± 6.7
Living situation		
Independent, with partner	76 (72.3%)	26 (36.6%)
Independent, alone	22 (20.1%)	34 (47.9%)
Institutionalized	3 (2.9%)	6 (8.5%)
Other	4 (3.8%)	5 (7.0%)
Level of education		
Low education	23 (21.9%)	23 (32.4%)
Medium level education	31 (29.5%)	24 (33.8%)
Higher education or university	49 (46.7%)	24 (33.8%)
Cardiovascular diseases		
Coronary disease	58 (55.2%)	13 (18.3%)
Heart failure	23 (21.9%)	8 (7.6%)
Atrial fibrillation	24 (22.8%)	9 (8.6%)
CVA/TIA	32 (30.5%)	17 (23.9%)
Peripheral artery disease	9 (8.6%)	3 (4.2%)
Cardiovascular risk factors		
Alcohol consumption in units/week	4 (0-8)	3 (0-6)
Smokers or ex-smokers	74 (70.5%)	31 (43.7%)
Hypertension	76 (72.3%)	46 (64.7%)
Hypercholesterolemia	42 (40.0%)	28 (39.4%)
Glucose	9.9 ± 4	10.0 ± 4
BMI in kg/m2	28 ± 5	27 ± 6
Systolic BP in mmHg	147 ± 22	148 ± 23
Diastolic BP in mmHg	80 ± 11	82 ± 10
LDL in mmol/l	1.9 ± 0.9	2.3 ± 1.0
HDL in mmol/l	1.2 ± 0.4	1.5 ± 0.5
eGFR CKD-EPI in mL/min/1.73 m²	65 ± 17	62 ± 18
Statin use	71 (67.6%)	41 (57.7%)
Anticoagulation		
DOAC/VKA	29 (27.6%)	12 (16.9%)
Platelet inhibition	53 (50.5%)	27 (38.0%)
Gait speed in m/s	0.86 ± 0.31	0.72 ± 0.30
Cognitive diagnosis		
SCD	11 (10.5%)	10 (14.0%)
MCI	44 (41.9%)	13 (18.3%)
Dementia	50 (47.6%)	48 (67.7%)

Data are presented as mean ± SD, n (%) or median [interquartile range]. Differences were tested with independent t-test for continuous variables and chi-square tests for categorical and for not normally distributed continuous variables.

Abbreviations (BMI=body mass index, BP=blood pressure, CVA=cerebrovascular accident, DOAC=Direct Oral Anti-Coagulant, GCA = global cortical atrophy, MCI = Mild Cognitive Impairment, MTA = Medial Temporal lobe Atrophy, TIA=transient ischemic attack, SCD = subjective cognitive decline, VKA=Vitamin K Antagonist, WMH = White matter hyperintensities)

Suppl table 2. The presence of interaction of diabetes and sex on an additive scale, quantified by RERI (Relative Excess Risk due to Interaction) analysis.

	RERI (95% CI)
Atrophy	0.45 (-0.64 – 1.46)
WMH	0.01 (-0.89 – 0.86)
Microbleeds	0.20 (-0.57 – 1.09)
Lacunes	0.48 (-0.87 – 1.84)

A RERI above 0 is indicative of an interaction of female sex and diabetes on the outcome. RERI below 0 is indicative of a positive interaction between male sex and the outcome.
WMH = White Matter Hyperintensities
